# Impact of emotion-laden acoustic stimuli on group synchronisation performance

**DOI:** 10.1038/s41598-023-34406-2

**Published:** 2023-05-01

**Authors:** Marta M. N. Bieńkiewicz, Stefan Janaqi, Pierre Jean, Benoît G. Bardy

**Affiliations:** EuroMov Digital Health in Motion, Univ. Montpellier IMT Mines Ales, Hérault, Montpellier, 34090 France

**Keywords:** Human behaviour, Applied mathematics, Auditory system, Emotion, Motor control, Oculomotor system, Sensorimotor processing, Social behaviour, Social neuroscience, Visual system

## Abstract

The ability to synchronise with other people is a core socio-motor competence acquired during human development. In this study we aimed to understand the impact of individual emotional arousal on joint action performance. We asked 15 mixed-gender groups (of 4 individuals each) to participate in a digital, four-way movement synchronisation task. Participants shared the same physical space, but could not see each other during the task. In each trial run, every participant was induced with an emotion-laden acoustic stimulus (pre-selected from the second version of International Affective Digitized Sounds). Our data demonstrated that the human ability to synchronise is overall robust to fluctuations in individual emotional arousal, but performance varies in quality and movement speed as a result of valence of emotional induction (both on the individual and group level). We found that three negative inductions per group per trial led to a drop in overall group synchronisation performance (measured as the median and standard deviation of Kuramoto’s order parameter—an index measuring the strength of synchrony between oscillators, in this study, players) in the 15 sec post-induction. We report that negatively-valenced inductions led to slower oscillations, whilst positive induction afforded faster oscillations. On the individual level of synchronisation performance we found an effect of empathetic disposition (higher competence linked to better performance during the negative induction condition) and of participant’s sex (males displayed better synchronisation performance with others). We believe this work is a blueprint for exploring the frontiers of inextricably bound worlds of emotion and joint action, be it physical or digital.

## Introduction

The ability to interchange with other people is a cornerstone of human social skills, weaving the functional fabric of societal cohesion and ability to act together. The ability to detect, predict and align with the actions of others, creates the uniquely human sense of agency and adequacy. The links between joint movement and emotion are established early in human development through successful (behavioural) mirroring of emotional arousal by an infant’s primary care-takers^1^. It is the care-taker that helps the baby to organise its displayed emotional state (i.e., crying, hiding) into a second-order cognitive construct (i.e., ”you are sad”, ”you are scared”)^2,3^. Repeated and reinforced mirroring by parental figures allows children to develop a sense of ’self’ (identity), and learn how to regulate emotional states on their own (restore allostasis). In adulthood, signalling emotions (e.g., friendliness) and reading the state of others, supported by societal reinforcement (feelings of affiliation^4,5^) and hormones such as oxytocin^6,7^, feeds into our cognitive expectations of what is going to happen in our direct environment in the nearest future (i.e., threat). And yet, our understanding of how that happens in a heartbeat, across different modalities (such as movement) that might convey information about emotions and intentions^8^, has largely escaped the modern interdisciplinary science.

In this context, our aim is to understand the socio-motor embodiment of emotion during synchronous joint action performance in a small group of people (a foursome). Synchronisation in the animal world has been explored via measurement of perceptual-motor coupling and behavioural synergies over time^9^. Humans demonstrate patterns of fluid synchronisation, across proximal and distal parts of a body system (be it posture, limbs, gaze, voice, respiration, skin conductance), flowing in and out of locking patterns for phase and frequency between agents^10–12^. Fluctuations between being in- and out- of synchrony^12^ are reported to operate multi-modally across behavioural and physiological synchrony (encompassing the autonomic nervous system, inter-brain synchrony and hormonal synchrony). On the mathematical level, plethora of studies have used one of the two models to compute the ’order parameter’ (a measure of the degree of synchrony between phases of oscillatory signals) during interaction between agents: the Kuramoto model (capturing in-phase coordination) or the Haken-Kelso-Bunz (HKB) model (capturing both in phase and anti-phase coordination and therefore bi-stability)^13^. Although both models approach synchrony without the need for a central controller such as an internal clock, neither of them have included so far the layers of movement qualities that convey the social information nor have looked into the impact of emotions on synchronisation performance^4^. Pioneering work on joint drumming showed that physiological and behavioural synchrony was predictive for the perceived cohesion among participants^14^. Links between physiological synchrony and cohesion have also been reported in crowds during live sport event viewing^15^, and group decision making tasks^16^, cementing the stance that social synchronisation is of paramount importance for relational emotions (i.e., affiliation, likability, admiration or attractiveness) in the domains of sport, art and education^10^, with profound social consequences^4,5^.

Studying emotion in the lab has become a major challenge in neuroscience and in affective computing, due to (at least) three pitfalls. Firstly, the very nature of emotion is elusive not only for external observers, due to its transiency and being constructed internally by agents^17^, but at times the agent itself might not be fully aware of its emotional state (and therefore not able to give accurate self-report). In our study, we used a high frequency motion recording to capture perturbations in the execution of a simple motor task (due to emotion-laden acoustic stimuli), and also collected appraisals of the valence and arousal of the perceived stimulus by each participant (not their self-state).

Secondly, the embodiment of emotion in movement (and body postures) and bio-signals (physiological arousal and rhythms) is subject not only to individual variation (in behavioural and physiological expression, or duration of arousal), but also to the context of culture and previous experience, which is hard to control for^18^. In our study, we looked for the effects of sudden, acoustic emotion induction on the back-and-forth finger movement during a synchronisation task. We used a validated sound battery (which admittedly does not remove the personal variation, which we controlled for using the SAM assessment^19^). We also accounted for the emotional state prior to the start of experiment using the PANAS questionnaire^20^.

Finally, the majority of experiments observe an individual removed from any naturalistic scenario (i.e., detached from a simple social setting), therefore hindering the natural milieu of emotions (where signalling of emotion plays important function). Notably to date, a plethora of emotion and movement research conducted experiments based on ’enacted’ emotions i.e.^21^, which do carry hallmarks of actual emotional expression, but are often exaggerated (especially if actors are the experimental subjects)^4^. And while there are other studies that involved autobiographical recall^22^, or induction with pictorial/video stimuli in psycho-physical context, but without movement data^23^, these methods are weak in terms of induction to produce a pronounced emotional arousal. In our study, participants entered a a social setting of playing a collaborative game, with a common, shared goal, where emotion was induced by acoustic stimuli. Whilst not a fully naturalistic setting, this allowed for ecologically valid induction of emotion and immersion of individuals in the physical presence of others, who are also engaged in the same motor task with a shared goal.

In this article, we introduce a research paradigm for exploring the impact of emotion-laden acoustic stimuli on a group synchronisation task in a digital environment. Here, we present a multi-agent experimental space, which allowed participants to perform the joint action task in their respective booths, without any direct eye contact, whilst sharing the same physical room with other players. To achieve this, we have built upon the previously established Chronos interface for disembodied socio-motor synchronisation^24–26^. Chronos was designed to afford the virtual version of the simplified mirror game for multiple agents interacting in real time^27^ via a network-based digital platform (visual display and movement capture device). With this interface, participants are able to align their movement (represented on the screen as a moving circle) with the movement of others. This methodology allowed us to control and reduce the limb motor signal to one horizontal dimension, where each participant’s arm movement was represented by a moving colour-coded circle (providing a flow of perceptual information, crucial for achieving synchronisation in humans^10^). In the original version of the mirror game paradigm, participants create interesting, joint movement together. Here, we asked participants to move together, back-and-forth in an oscillatory fashion, to create a unified movement.

Emotional induction for each participant consisted of a pseudo-randomised set of acoustic signals chosen from the IADS-2 sound battery^28^ and displayed during consecutive trials (see the Supplementary Information Tables S1 and S2). Although The International Affective Digitized Sounds (IADS-2)^28^ have been validated in multiple studies^29,30^, we aimed to evaluate the feasibility of using those sounds during a joint action task or any motor task at all (which we have not found in the literature to date). Acoustic stimuli unlike their visual counterparts (i.e., International Affective Picture System^31^), do not consume the same attention resources and can be delivered to each individual in a discrete fashion (via headphones^32,33^) which can be advantageous for group scenarios in which multiple agents are concerned.

Our research questions (RQ) were the following:

1. Are participants able to adequately perceive the valence and arousal of the sounds selected from the IADS-2 battery during the task?
2. Does exposure to emotion-laden acoustic stimuli during socio-motor synchronisation task impact group performance metrics (median and standard deviation of Kuramoto’s order parameter, and time spent in higher synchronisation bands)?
3. Does exposure to emotion-laden acoustic stimuli during socio-motor synchronisation task impact participants’ speed of movement? Based on previous research^34–36^ we expected to observe a trade-off of positive and negative induction with the speed of movement (with positive emotion affording faster oscillations and reversely, negative inductions slowing down the performance).
4. Are people who report higher sensitivity to emotions displayed by others more empathically tuned in to others, and do they perform better in joint synchronisation tasks (measured by an Individual Synchronisation Index derivative of group Kuramoto’s order parameter)?

## Results

Participants completed 54 trials of the group synchronisation task (18 per emotion condition: negative, neutral, positive; see Table S2 for illustration of the trial run across participants). Between trials, participants were asked to appraise both the valence and arousal of the sound stimuli. To assess the performance per group per trial we computed Kuramoto’s order parameter based on the finger movement data of all participants interacting. In subsequent steps, we computed standard deviations of this parameter, and other metrics such as Time in synchrony (i.e. the time for which participants maintained synchronisation in each order parameter band) and Time to reach synchrony (i.e. the time required to cross the threshold of each order parameter band). In addition, we computed the number of cycles completed per group per trial in order to capture the speed of performance during synchronisation. For the in-depth analysis of individual contributions to the group’s synchronisation performance, we extracted an Individual Synchronisation Index which shows the closeness of each agent’s phase to the order parameter of the group at each time instance. This allowed us to look into effects of sex and empathetic ability on performance. We also measured the number of cycles completed by each participant.

### Validation of emotional induction

**Figure 1:**
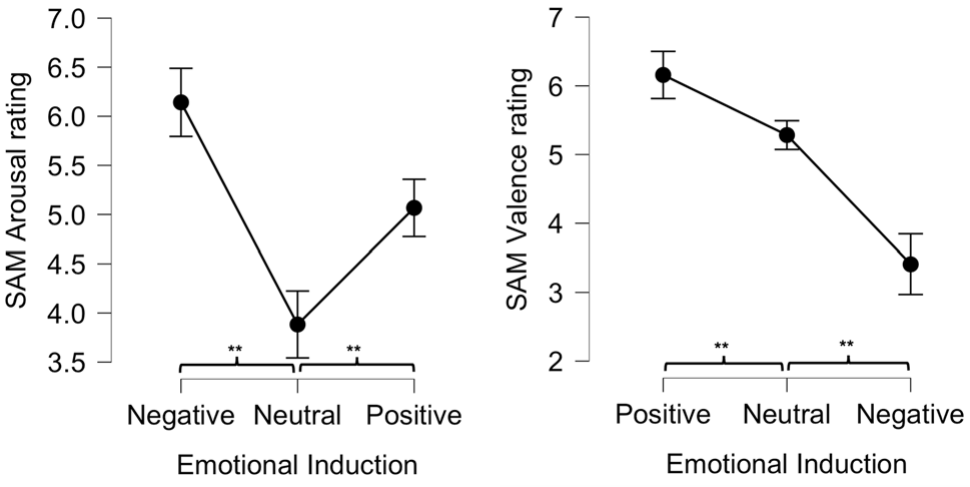
Emotion Induction—valence and arousal ratings obtained in the tested sample. Participants appraised the received acoustic stimuli as intended for arousal (higher values for negative and positive stimuli than neutral) and valence (high scores—positive, low scores—negative). Statistical significance for main effect of emotional induction on the arousal/valence SAM rating is marked with asterisks (**p<0.001). Error bars denote 95% confidence intervals.

We measured participants’ appraisal of the emotional arousal and valence of the stimuli using Self-Assessment Manikin protocol^19^. In the tested sample, we found that participants adequately appraised the stimuli received, whilst being immersed in the group motor task. To conduct inferential statistics we took the median value (from 18 trials per condition) for valence and arousal separately per emotional condition per participant. Repeated measures ANOVA showed a large effect of the emotion induction on the SAM valence ratings F(2,104) = 191.84, $\eta ^{2}$ = 0.78, large effect size, with all emotion inductions being significantly different from each other (*p*<0.001) (see Fig. 1). For the arousal ratings on the SAM F(2,104) = 44.91, $\eta ^{2}$ = 0.46, large effect size, all emotion inductions also differed significantly from each other (*p*<0.001) (see in the Supplementary Information Tables S3 and S4 for overview of the descriptive statistics). Neither PANAS^20^ positive or negative scores, or overall score on Emotional Contagion scale^37^, explained the SAM scores for the Emotional Induction condition with logistic regression (p>0.05), we have not found effect of the run order (see Table S2) on the SAM ratings (p>0.05).

### Behavioural analysis of group performance

**Figure 2:**
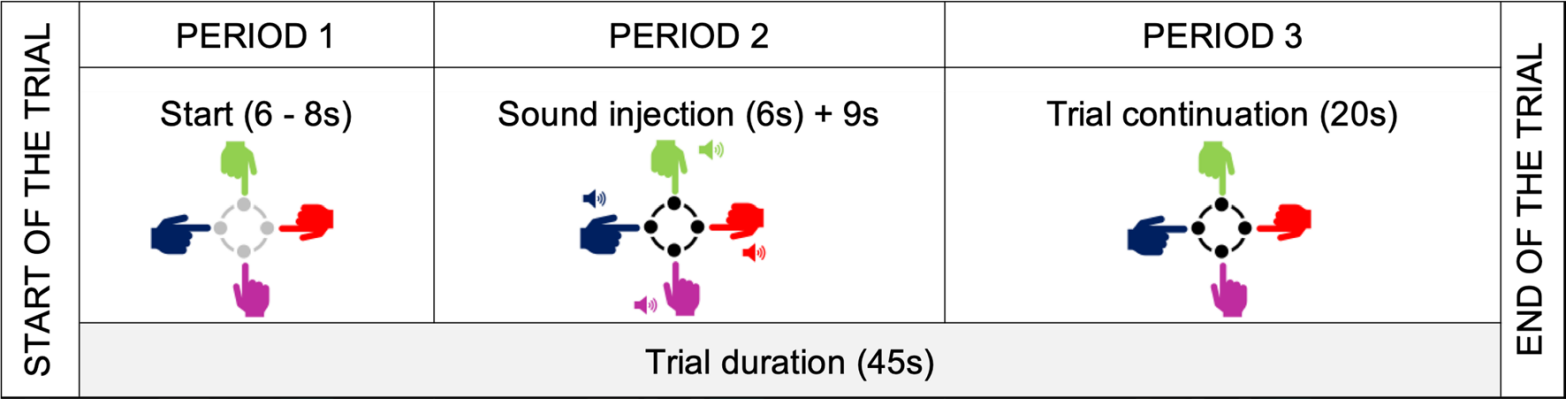
Three segments extracted from the trials. Segmentation of the trials allowed to understand the impact of emotional induction immediately and after short passages of time; Period 1—Duration of 6 sec prior to onset of the sounds; Period 2—Sound duration (6 sec) plus 9 sec time window (the length of time that participants were able to maintain synchronisation after perceptual interruption was estimated as approximately 7s in a study using similar paradigm and apparatus (with mean of 8.81s for professional dancers and 6.26s and non-professional dancers)^10^; Period 3—Trial continuation - 15 sec post sound onset for 20 sec (continuation phase was considered by us a sufficient base to assess long-term differences caused by emotional induction). Fringes of the data frames were trimmed on each side of the trial to match the duration of the segments regardless of the sound onset delay—varied 6–8s across trials.

Our presentation of the data analysis is structured by the research questions targeting performance metrics, subdivided into Period 2 and Period 3 for each trial (see Fig. 2). To compute the effect of the emotional induction in a group, we aggregated individual inductions to two index values; number of negative and positive inductions per group per trial (with 3 negative or 3 positive inductions being the maximum number per trial). The numbers of negative and positive inductions per group per trial were entered into a Linear Mixed Model analysis as fixed factors (as numbers).

#### Synchronisation performance

**Figure 3:**
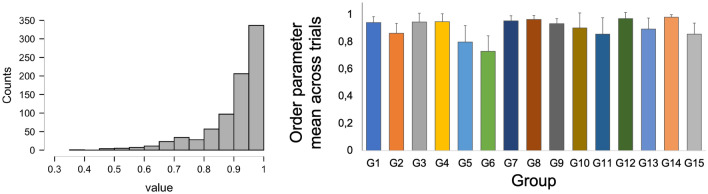
Histogram of median values of order parameter per trial for all groups and means of median values of order parameter across all trials presented per group. Error bars denote standard deviation from all trials.

To quantify synchronisation between agents, the Kuramoto order parameter was computed from spatio-temporal data following the methodology and scripts developed in^10^. The trial was subdivided into three sections as depicted on the diagram below (see Fig. 2). Overall participants showed an aptitude for moving in synchrony with other members of their group in the digital environment (Median = 0.94; SD = 0.1, see Fig. 3 for the details about the distribution). There was a significant effect of the group on the median value per trial (F(14,795) = 46.2, *p*<0.001,$\eta ^{2} = 0.44$, but no effect of the trial number, p>0.05). We found that the first trial across all groups was significantly worse in performance and created a bias in our data set (being a trial with three negative inductions per group) therefore we removed it for the behavioural part of the analysis, leaving the rest of 53 runs for all groups to remove distortion of the negative emotion effect due to the lack of training of the actual task.

#### Order parameter

**Figure 4:**
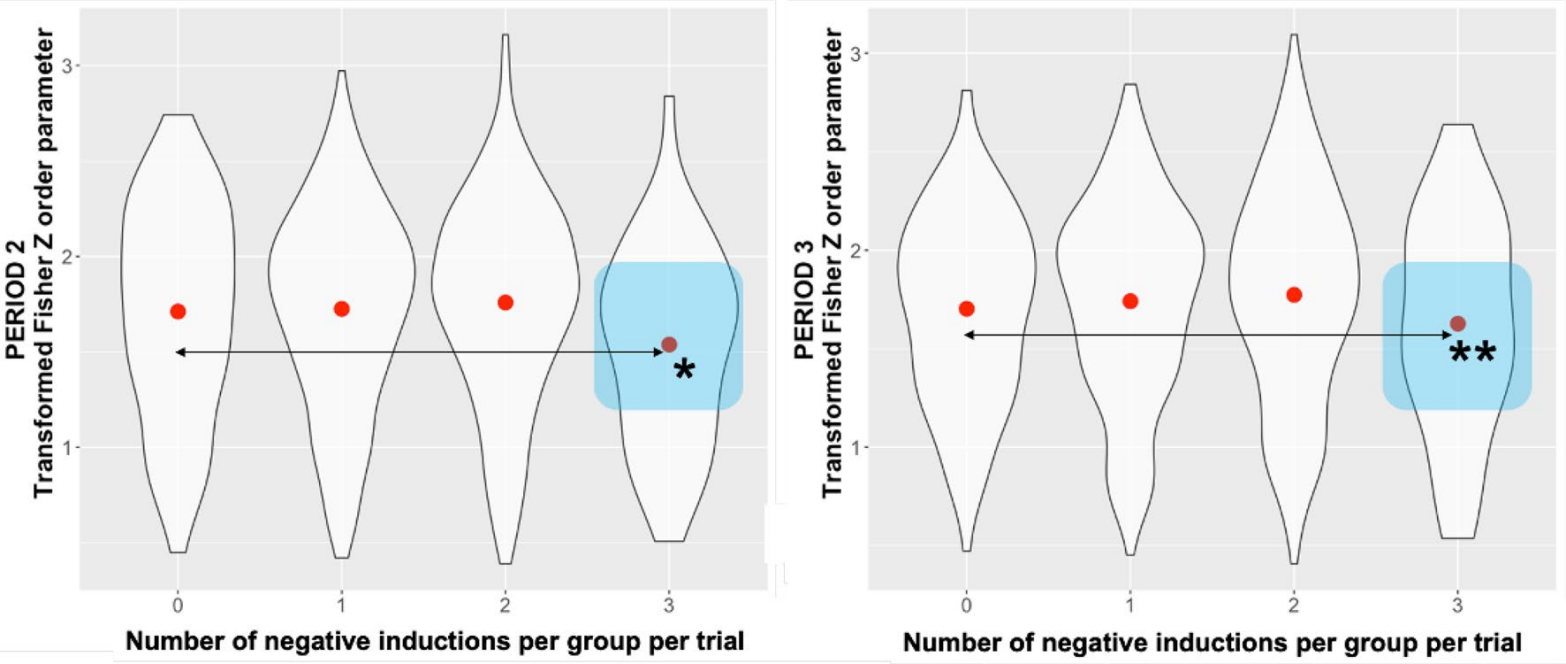
Average of median order parameter scores across number of negative inductions per group per trial. Asterisks denote significance level for the predictor, **(*p*<0.01), *(*p* = 0.01).

The median order parameter (computed as a measure of synchrony between agents) was converted to Fisher Z scores to produce a normalised distribution of the Kuramoto order parameter. Three negative inductions per group per trial caused a deterioration of synchrony per group per trial. For Period 2 (see Table S5 in the Supplementary material) we found an effect of three negative inductions per group per trial (M = 1.54, SD = 0.51), to none (M = 1.71, SD = 0.55), on the median Fisher Z score of order parameter: Beta = 0.06, CI = 0.02-0.11, *p*<0.01 (model: Marginal $R^2$/Conditional $R^2$ 0.008/0.528) and for Period 3 (see Table S6 in the Supplementary material) of three negative inductions per group per trial (M = 1.62, SD = 0.54), to none (M = 1.72, SD = 0.47), on the median Fisher Z score of order parameter: Beta = 0.06, CI = 0.01−0.10, *p* = 0.01 (model: Marginal $R^2$/Conditional $R^2$ = 0.007/0.579) (see Fig. 4).

Standard deviations of the order parameter were transformed to Fisher Z scores to produce a normal distribution to the LMM. For Period 2 (see Table S7 in the Supplementary material), we found an effect of three negative inductions per group per trial (M = 0.1, SD = 0.075), to none (M = 0.08, SD = 0.06), (Beta = −0.01, CI = −0.02–0.00, *p*<0.01) (model: Marginal $R^2$/Conditional $R^2$ = 0.01/0.48), showing that during that period post injection of the sound perturbed the synchronisation and increased its variability. This effect did not carry over for Period 3 (see Table S8 in the Supplementary material), with only an effect of one positive induction per trial (M = 0.09, SD = 0.07), to none (M = 0.07, SD = 0.06), on the standard deviation of the order parameter, transformed to Fisher’s Z score (Beta = −0.01, CI = −0.02–0.00, *p*<0.01).

#### Time in synchronisation bands—H, M, W

**Figure 5:**
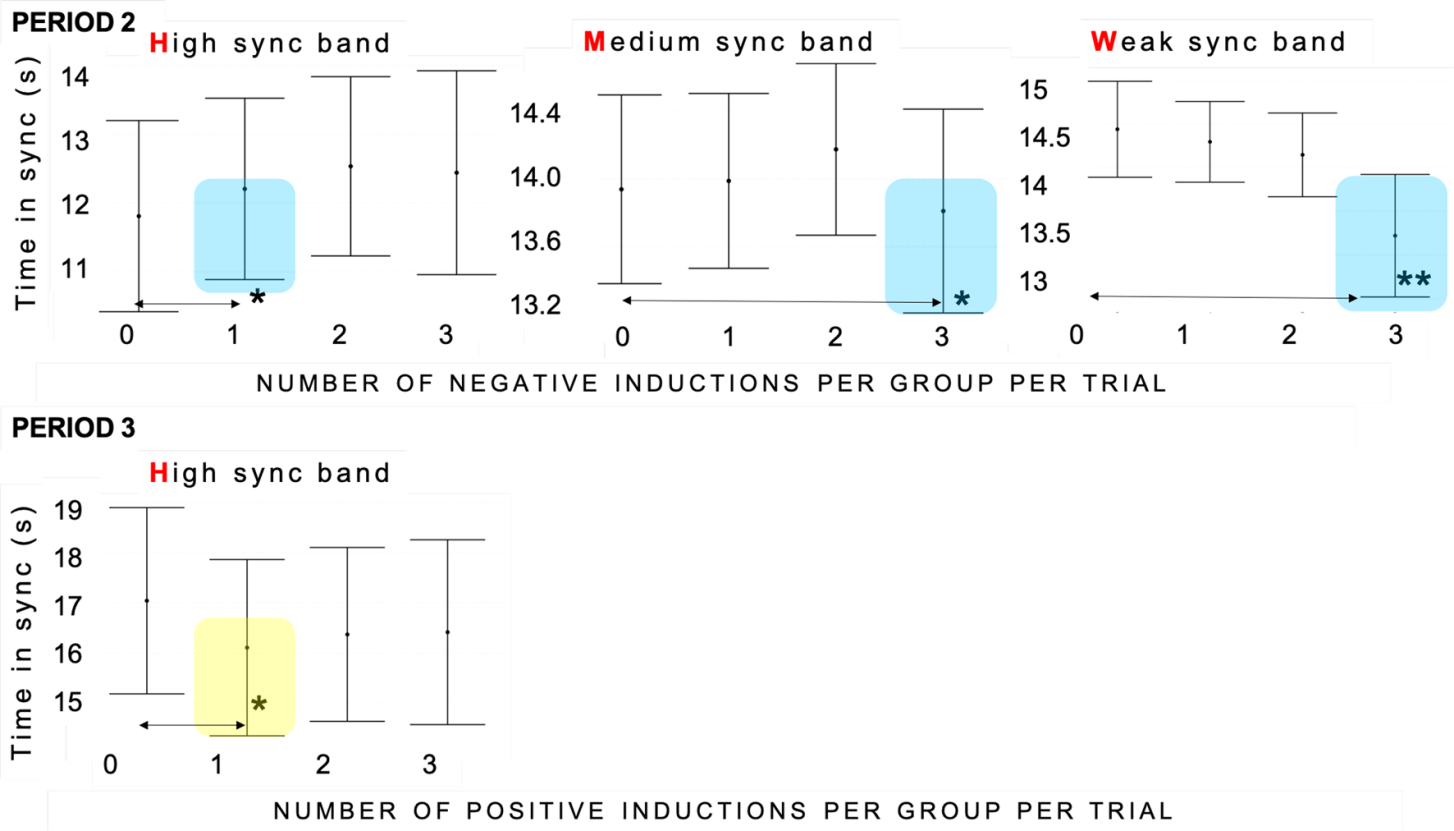
Effect plots for the time spent in synchronisation in over three order parameter bands. Significance levels for the predictor levels are denoted with asterisk(s) *(*p*<0.05) and **(*p*<0.001); and highlighted in light blue for the number of negative inductions per group per trial and light yellow for the number of positive inductions per group per trial. The whiskers denote 95% confidence intervals of the effects.

With this metric (see Methods section) we looked into the time spent in three bands of order parameter (see Fig. 11). This was defined as the overall time duration for which order parameter of the group per trial stayed above a given synchronisation threshold. In Period 2 (see Table S9 in the Supplementary material), we found an effect of one negative induction per group per trial on the time spent in High (H) synchronisation band [synchronisation range > 0.83] (M = 12.6, SD = 3.22), to none (M = 12.1, SD = 3.66), (Beta = −0.44, CI = −0.85–0.02, *p*<0.05) (model: Marginal $R^2$/Conditional $R^2$ = 0.005/0.523), suggesting that heterogeneous induction in a group, led to higher performance. Further, with more unified induction, we have found an effect of three negative inductions per group per trial for the time spent in Medium (M) synchronisation range [0.66–0.82](M = 14.02, SD = 1.52), to none (M = 14.21, SD = 1.32), (Beta = 0.20, CI = 0.05–0.35, *p* = 0.01) (Marginal $R^2$/Conditional $R^2$ = 0.009/0.423) (see Table S11 in the Supplementary material) and an effect of three negative inductions per group per trial for the Time spent in Weak (W) synchronisation range [0.5–0.66] (M = 14.85, SD = 0.33), to none (M = 14.92, SD = 0.19), (Beta = 0.04, CI = 0.01−0.07, *p*<0.01) (model: Marginal $R^2$/Conditional $R^2$ = 0.02/0.24)(see Table S12 in the Supplementary material). This finding are consistent with the findings of detrimental impact of three negative induction on the order parameter (previous section). Lower ranges of synchronisation bands were found in other studies to be sensitive to emotional induction^38^. In the continuation of the trial in Period 3, we found an effect of one positive induction on Time in H band per group per trial (M = 16.24, SD = 4.75), to none (M = 17.11, SD = 4.1) (Beta = 0.56, 0.00–1.11, *p* = 0.05) (model: Marginal $R^2$/Conditional $R^2$ = 0.005/0.51)(see Table S10 in the Supplementary material) (see Fig. 5). This suggests that in the continuation phase, heterogeneous induction has led to somehow prolonged worsened performance and is a finding to be further replicated.

#### Time to reach synchronisation bands—H, M, W

**Figure 6:**
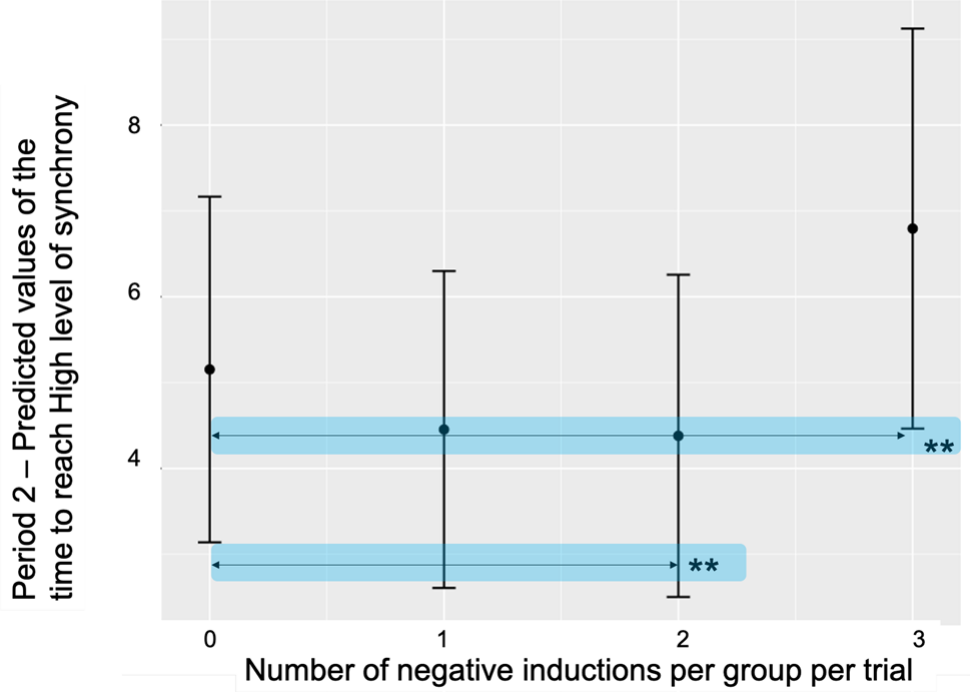
Predicted values for the model of Time to reach High levels of synchrony in Period 2 based on the number of negative inductions per group per trial. The whiskers denote 95% confidence intervals of the effect. Significance levels for the predictor are denoted with asterisks **(*p*<0.001).

Time to reach synchronisation bands was computed as the time to surpass a given threshold (see Fig. 11) of synchronisation for the first time in the selected time window for analysis and stay within for a given minimum duration. For the immediate Period 2 (see Table S13 in the Supplementary material) inclusive and following sound injection in the trial, we found an effect of the two negative inductions per group per trial (M = 4.55, SD = 6.09), to none (M = 5.05, SD = 6.44), (Beta = −0.74, CI = −1.34–0.13, *p*<0.05)—meaning that participants established the H ranges of synchronisation band faster, when two negative inductions were introduced in a trial, in comparison to trials without any negative inductions. This means that some heterogeneity in the emotional induction in the group per trial led to a faster and more precise temporal alignment (similarly to the finding in the previous section that one negative induction per group per trial was a predictor for more time spent in H synchronisation band). However, in line with previous findings, three negative inductions per group per trial (M = 6.99, SD = 7.05), compared to none, led to longer time to achieve H synchronisation bands (Beta = −0.82, CI = −1.47–0.16, *p* = 0.01) (model: Marginal $R^2$/Conditional $R^2$ = 0.01/0.33). This means it was harder for the group to reach higher ranges of synchronisation when three people were induced with negative emotion-laden stimuli (Beta = −0.82, CI = −1.47–0.16, *p* = 0.01) (model: Marginal $R^2$/Conditional $R^2$ = 0.01/0.33). None of the aforementioned effects carried over the rest of the trial (Period 3) (see Fig. 6). For the M synchronisation and W synchronisation bands no fixed effects were identified.

#### Number of cycles

We then analysed how many synchronisation cycles participants were able to achieve within each period (alternatively it could be computed and represented as i.e., mean cycle duration). This was computed as mean across participants number of times the hand (number of maxima) achieved its maximal position on X axis before reversal of movement direction.

We found a main effect of one negative induction (M = 10.28, SD = 2.46) in Period 2 (see Table S14 in the Supplementary material), to none (M = 10.55, SD = 2.58) per group (Beta = 0.43 CI = 0.27–0.59, *p*<0.001); two negative inductions (M = 10.04, SD = 2.2), to none, (Beta = 0.30, CI = 0.19–0.41, *p*<0.001); and one positive induction (M = 10.04, SD = 2.29), to none (M = 9.7, SD = 2.1), (Beta = −0.52, CI = −0.52 to −0.21, *p*<0.001); two positive inductions (M = 10.4, SD = 2.51), to none, (Beta = −0.20, CI = −0.31 to −0.10, *p*<0.001); three positive inductions (M = 10.81, SD = 2.55à), to none, Beta = 0.14, CI = 0.02–0.25, *p* = 0.02) (model: Marginal $R^2$/Conditional $R^2$ = 0.032/0.861).

**Figure 7:**
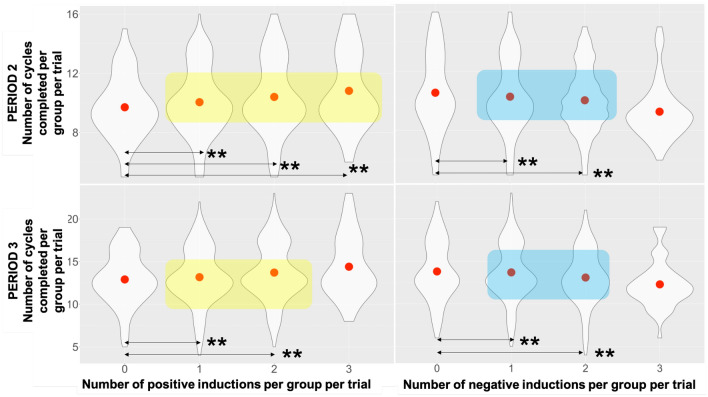
Violin plot for the number of cycles completed per Period 2/3 classified by the number of positive/negative inductions per group per trial. Asterisks denote significant predictor levels for the positive induction (yellow highlight) and for the negative induction (blue highlight). Significance levels for the predictor are denoted with asterisks **(*p*<0.001). **(*p*<0.001).

In Period 3 (see Table S15 in the Supplementary material), that means 15 sec post injection of the sound until the end of the trial, the effect of emotion induction remained with main effect of one negative induction (M = 13.68, SD = 3.39), to none (M = 13.8, SD = 3.41), (Beta = 0.45, CI = 0.24–0.66, *p*<0.001); two negative inductions (M = 13.08, SD = 3.18), to none, (Beta = 0.54, CI = 0.40–0.69, *p*<0.001); and one positive induction (M = 13.17, SD = 3.27), to none(M = 12.90, SD = 2.99), (Beta = −0.41, CI = −0.62 to −0.20, *p*<0.001); two positive inductions (M = 13.17, SD = 3.27), to none, (Beta = −0.32, CI = −0.47 to −0.18, *p*<0.001) (see Fig. 7) (model: Marginal $R^2$/Conditional $R^2$ = 0.031/0.867).

### Individual Synchronisation Index

**Figure 8:**
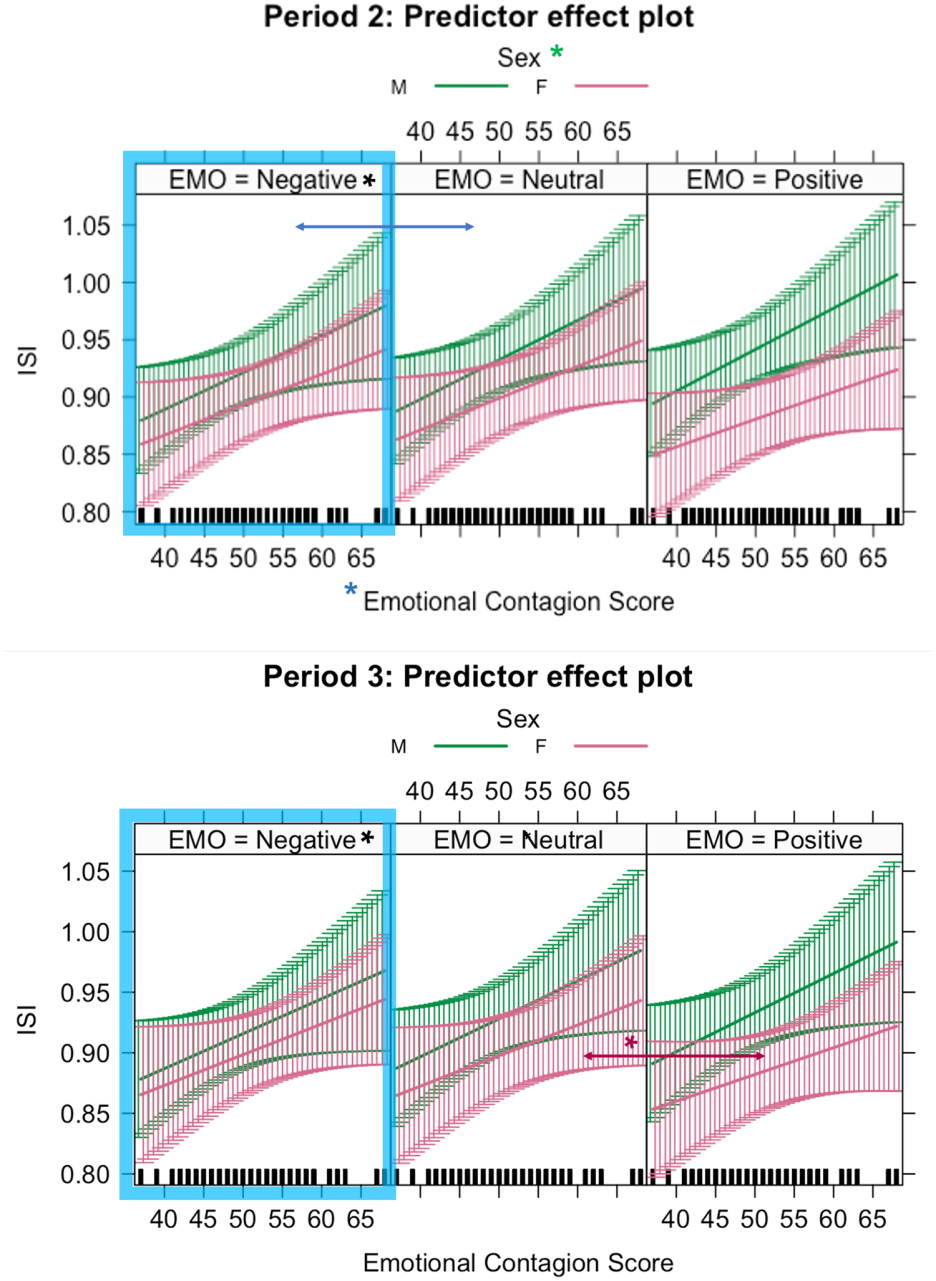
Effect plots for the Individual Synchronisation Index (ISI) and predictors: Sex of participants and score on the Emotional Contagion Scale. Top panel depicts Period 2 predictor effects on ISI. Blue box with black asterisk denotes the effect of the negative induction versus the neutral induction. Blue asterisk on the horizontal axis denotes the interaction of negative induction and Emotional Contagion Score. Female participants had on average lower ISI scores (green asterisk). Bottom panel depicts Period 3 predictor effects on ISI. Blue box with black asterisk denotes the effect of the negative induction versus the neutral induction. Magenta arrow and asterisk denotes the interaction of positive induction and female sex. The whiskers denote 95% confidence intervals of the effect. Significance levels for the predictor are denoted with asterisk, *(*p*<0.05) (see Tables S16 and S17 for reference).

For each participant per group we computed the Individual Synchronisation Index (ISI)—a mismatch between their phase and the phase of their group (the closer to 1 was the value, the lower the mismatch). There was a significant main effect of emotional induction on the ISI. As our distribution of residuals was heavy tailed we are reporting below the standardised parameters of the model. In Period 2 (see Table S16 in the Supplementary material), following immediately the sound injection, there was a fixed effect of negative emotion induction (M = 0.91, SD = 0.14), to neutral(M = 0.92, SD = 0.12) on ISI (std B = 0.05, std CI (0.01–0.09) *p*<0.05, and Sex (std B = 0.15, std CI (0.01–0.29), *p*<0.05) on ISI (males scoring higher (M = 0.93, SD = 0.13) than females (M = 0.9; SD = 0.14)(see Fig. 8).

We found also a fixed effect of the interaction between the negative induction and sex (std B = −0.05, std CI (−0.09–0.01), std *p*<0.05), and a fixed effect of interaction between the negative induction and Emotion Contagion^37^ score (std B = −0.05, CI (−0.10–0.00), std *p*<0.05) (model: Marginal $R^2$/Conditional $R^2$ = 0.055/0.39) (see Fig. 8).

Similarly for Period 3 (see Table S17 in the Supplementary material), there was a persistent fixed effect of negative emotion induction (M = 0.91, SD = 0.13), to neutral (M = 0.92, SD = 0.12) on the value of ISI (corrected values: std Beta = 0.05, std CI (0.01 to −0.09), std *p* = 0.01), showing that negative emotion induction disrupts the ability to synchronise with others surpassing the period of 9 sec post the end of the emotion induction. There was also an interaction between positive induction with sex (females: neutral (M = 0.97, SD = 0.02) versus positive (M = 0.96, SD = 0.02)) (corrected values: std Beta = −0.07, std CI (−0.15 to −0.00), std *p* = 0.05), (model: Marginal $R^2$/Conditional $R^2$=0.048/0.437).

## Discussion

Humans are incredibly skilled at synchronising with each other. In our study, we tested a sample of 60 adults, assigned to 15 mixed-sex groups of four, during a digital version of the disembodied movement synchronisation task. As the first objective of the study, we aimed to validate the feasibility of using sounds from the IADS-2 (RQ 1.) to induce positive and negative emotions during a motor task performance. We found that the IADS-2 stimulation elicited appropriate perception of emotional valence and arousal in the tested sample (see Fig. 1). On average, this method appears valid to be introduced in experimental scientific protocols, as sound is a portable and powerful vehicle of emotion-laden information^33^, which can be administered when participants are attending to other visual stimuli. A previous study using IADS-2,^39^ demonstrated that female participants reacted with changes in their heart rate variability (HRV) to the valence and arousal in a consistent pattern. Another repeated measures study using the same stimuli^40^ showed that those HRV reactions were not found to be susceptible to habituation after a one week interval. These authors also investigated the skin conductance and zygomatic muscle activity, and showed a consistent pattern of psychophysiological responses for IADS-2 stimuli, thus validating the perceptual self-report data from original report^28^. Unlike the psycho-physical paradigm that is usually paired with IADS-2 battery, in the current study, participants were engaged in a joint motor task. The problem with emotional induction in the lab setting was briefly discussed in the introductory part of this article and referred to a review article on the subject^4^. We believe that the current report is an important example to show the possibilities of using emotion-laden acoustic stimuli; as emotional induction in movement studies. Although we have noted some degree of variation in appraisal of the stimuli, for example sounds that aim to be relatively neutral (low or medium arousal, medium valence) might evoke an idiosyncratic reaction due to personal association linked to acoustic memory, we believe it can be controlled via the SAM psychometric report post exposure to the stimuli.

Our core research objective (RQ 2.) was to explore the impact of emotions on group synchronisation performance. Our findings show that synchronisation is resistant to fluctuations in individual emotions (see Fig. 3), but its quality deteriorated with a higher number of negative inductions per group per trial. In our data set, we have observed that group synchrony deteriorated with three negative inductions per group per trial. This was measured as both the drop in the order parameter value (Fisher Z-transformed; see Fig. 4) as well as the increase in variability (measured as Fisher Z-transformed standard deviation of order parameter) during Period 2 (15 sec immediately after onset of acoustic stimuli). Similarly, in terms of time spent during Period 2 in different synchronisation bands (characterised as high—H, medium—M and weak—W coupling, see Fig. 11), one negative induction was found to be a predictor for more time spent in H band (in comparison to none), and three negative inductions per group per trial were predictors of less time spent in bands M and W during trials (see Fig. 5). W band was recently identified by another study^38^ as being sensitive to the impact of emotional induction during a joint improvisation task. For Period 3 (continuation of the trial 15 sec after emotional induction): a drop in the time spent in the H band was noted for one positive induction, which suggests that the group behaviour deteriorated when the induction was not homogeneous across the group (such as two or three positive inductions, which might be linked to the speed of performance). This effect needs further replication to investigate the underlying mechanism. In Period 2 (immediate induction period and 9s after), we identified fixed effects of two and three negative inductions per group per trial on the time to reach H level of synchronisation in comparison to none (see Fig. 6). More time was needed with the three negative inductions per group per trial in comparison to none to reach H band as a group (no fixed effects were found in Period 3, or for time to reach synchronisation in medium—M or weak—W bands). With two negative inductions, the time to reach H band was shorter than no negative induction, which is a unexpected effect, pointing again at the complexity of inter-agent the synchronisation performance. Based on the analysis of the pace of performance (RQ 3.), measured as number of cycles achieved during Period 2 and Period 3 (see Fig. 7), we found that positive induction (one, two and three per group per trial) enabled gradually faster group movement, while negative inductions (one and two per group per trial) led to slower performance (lower number of cycles achieved). In Period 3, those fixed effect persisted for: one and two, both negative and positive inductions per group per trial. Therefore, we suggest that slower performance led to faster stabilisation of performance in higher synchronisation bands until the majority of the group was induced with negative emotions, the advantage disappears and led to prolonged stabilisation of synchronisation.

This work is a novel contribution to the body of literature, in terms of impact of emotions of group synchronisation performance. Previous research on motor timing in a solo performance paradigm reported speeding up of performance following negative emotional induction^34–36^. Our work demonstrates that three negative inductions per group per trial worsened the group synchronisation performance and slowed down the performance. Positive induction was associated with faster oscillations in both immediate time window during and post induction (Period 2), and in the continuation of the trial (Period 3). Therefore it seems that in individual motor tasks, the reaction to emotional induction might vary from the group setting. Group synchronisation performance did not linearly worsen as the number of negative inductions increased from one to three. The observed group pattern was inconsistent across synchronisation bands for one and two negative inductions. This calls for more research to further examine both the homogeneous and heterogeneous emotional induction of agents.

Noteworthy, looking at the ISI—an Individual Synchronisation Index (RQ 4.), we managed to dissect the difference in synchronisation performance between male and female participants and empathetic disposition. We found that negative induction (compared to neutral) was a predictor for decreased performance in both Period 2 and Period 3. In addition, in Period 2, we found an effect of sex, with females having lower ISI than male participants. We found an effect of Emotional Contagion score, for negative induction on ISI (higher empathetic disposition for Emotional Contagion led to higher ISI performance), shedding light on this new finding that participants who self-reported to be more attuned to the emotional expressions of others, were also better at staying in synchrony with others immediately after the negative emotional induction. This small and transient effect is interesting to note for further investigations, with reference to the evidence presented in the introduction suggesting parallel development of empathy and synchronous socio-motor interaction during human development^4^. We also found a difference in ISI between male and female participants (with male participants having on average higher ISI than females)(see Fig. 8). We are not aware of any studies reporting differences in synchronisation performance between sexes, but previous research reported (1) advantage in males since adolescence in the motor timing task (attributed to faster motor learning)^41^ and (2) enhanced spatio-temporal performance of males in a visuo-oculo-manual motor task (attributed to faster prediction process coupling target motion to a tracking hand)^42^. In a recent study^43^ a novel piece of evidence emerged that heterosexual women report higher attraction to males who are ’super synchronisers’ during naturalistic socio-motor interaction (measured as level of coupling in body alignment), which suggests perhaps that there is a innate advantage in males, who are more attuned to sensori-motor signals. This interaction effect reaffirms the need for future experimental work to validate the main effects reported here, and to clarify the nature of the interaction between empathetic disposition and synchronisation performance, independent of emotional induction.

We acknowledge that in this setup, the evoked emotions did not hold any personal context for the participants. The self-reports analysed here are perceptions of external stimuli. Be this as it may, the situation holds a lot in parallel to those in real-life (e.g., walking with a group of friends and being the only person who sees a disturbing scene on the sidewalk; hearing your pet cry in the room next door when one is working in the digital space in their home office; cycling in a group and hearing a car horn). As the enmeshment between digital and physical worlds progresses with time, further investigations into the impact of emotion arousal on sensori-motor interaction should follow in both separate (digital versus physical) and hybrid contexts. With the recent COVID-19 pandemic experience, social distancing made further exploration of socio-motor interaction and embodiment of emotion in the context of research even harder. As much as the digital world opened new doors to border- and boundless, time independent communication (like sending video or audio messages to your friends), it shut the door to full and rich human contact, with soft elements and nuances missing from the flow of information. This lack of rich interpersonal relay leaves digital exchanges tiresome in the long run, leaving our brains unsatisfied with futile attempts to understand and predict other people’s behaviour. For example, the asynchronous nature of email exchange or the misreading of people’s actual state during video calls^44,45^. Even a millisecond-long lag in the flux of information (or between its modalities) can lead to disruption of turn-taking and can ruin the fluidity and spontaneity of interaction between agents. Those, among many other idiosyncrasies of digitally nested interactions, are yet to be fully investigated. Limitations of digital (versus physical) contact that emerged during the COVID-19 pandemic brought to everybody’s attention a need to create digital environments enriched with emotional qualities. Creating digital environments with communication flow that is as meaningful as interpersonal has contact became of paramount importance for future research and development in this field. We propose this paradigm as a novel pathway for exploring the impact of emotional arousal on group performance and individual movements within that context.

## Methods

### Participants

Participants were recruited for the study by a snowballing strategy from the student and staff pool of University of Montpellier. Ethical approval was granted by the EuroMov ethical committee (IRB EuroMov #2005A) in accordance with the Declaration of Helsinki. Written informed consent was obtained from all subjects after receiving an explanation of the study. 60 individuals (28 females) participated and were randomly assigned to 15 groups of 4 people (mixed sex and age: M = 26+/- 6 years). All volunteers were neurologically healthy and had normal or corrected to normal vision and normal hearing, 3 were left-handed (measured with Edinburgh Oldfield Handedness questionnaire^46^). Since the vision of other players in the group was obstructed by booths’ walls, it excluded the need to control within the group for social cues such as the acquaintance level between participants, age, or gender. Each of the participants followed a strict social distancing protocol for COVID-19 prevention.

We have estimated a priori the minimal sample size to be N = 15 for the fixed effect of emotional induction on the synchronisation performance per group per trial. We used G*Power by using the Cohen’s^47^ suggested benchmark criteria for partial $\eta$ $^{2}$ for medium effect size = .06, we set the effect size of f = .25, $\alpha$ = .05 and power of the test (1-$\beta$) = 0.8, 3 levels of factor, 18 measurements per factor. For the individual performance (effect size of f = .25, $\alpha$ = .05 and power of the test (1-$\beta$) = 0.8, 3 predictors) the minimal sample size was calculated as 48 participants.

### Apparatus

Chronos is an open-source software that provides a digital, disembodied platform for motor interaction. Movement of all participants is translated in real time from their personal space to a gaming display of controlling and moving colour-coded circles on their respective screens (see^24^). The positional data of their index finger (of the preferred hand) was recorded with the LEAP motion at a sampling frequency of 55Hz. In this experiment we fixed the multiplayer Chronos typology to ”complete” (see^10^) without a predesignated leader (meaning that all participants were seeing each other’s movement as illustrated on Fig. 10).

**Figure 9:**
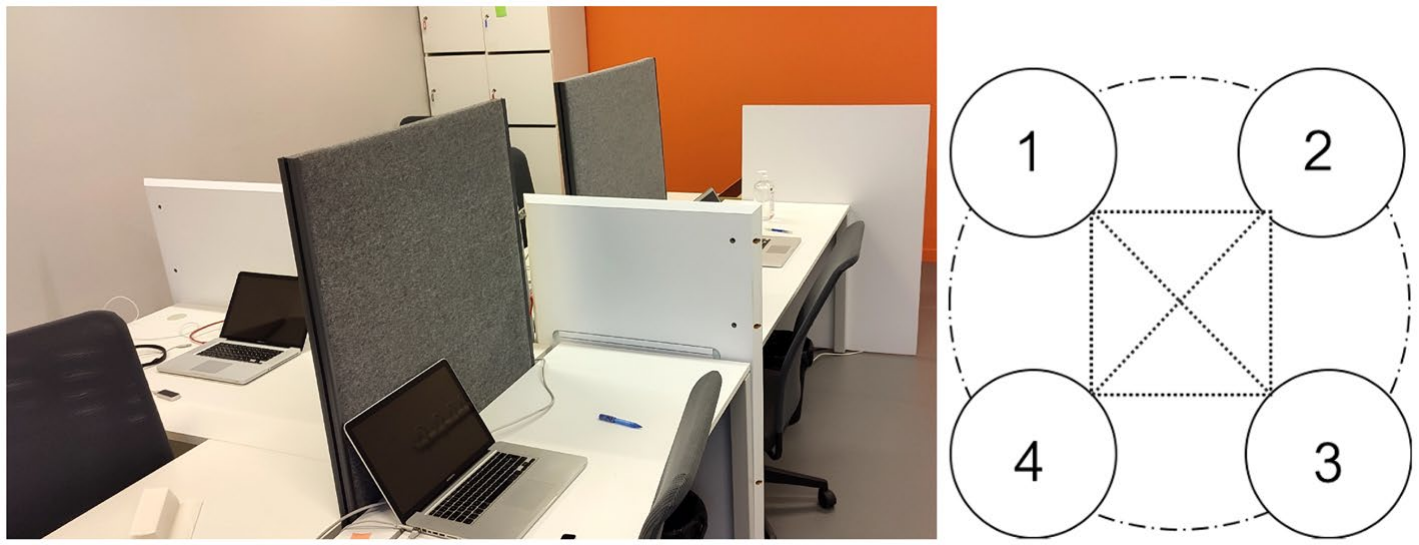
Laboratory setup—player booths. Left panel depicts the experimental room with the shields between participants to prevent view and real-time identification of the other members of the group. Right panel shows the complete^10^ coupling structure between participants using the digital display of moving, colour-coded circles.

### Procedure

The use of the LEAP motion sensor was demonstrated by the researcher at the demonstration booth - the required finger movement with preferred index finger and the optimal capture position and distance (index finger hovering between 10 and 20 cm in a vertical/diagonal line from the LEAP motion sensor). Participants were instructed to complete one solo trial as a practice run, with white noise being played in the background. After that, participants were asked to take a seat in their own booth—in front of their own computer display and capturing device, at 1.5m distance with direct visual contact prevented by shields (see Fig. 9 for the schema of the set up). Just before the launch of the multiplayer game participants were invited to fill in two short questionnaires - PANAS^20^ (to assess their current emotional state prior to the experiment (French validation,^48^), and The Emotional Contagion scale to assess their empathetic ability (^37^, adapted by^49^). PANAS is a 20-item self-report scale where participants use a 5-point Likert scale to describe their current mood using adjectives on a list of positive and negative emotions^50^. PANAS provides two outcome scores for: (1) positive affect; (2) negative affect. The Emotional Contagion Scale is a 15-item self-report scale where participants use 5-point Likert scale to describe their susceptibility to ”catching” the emotional states of others (^37^^49^). The outcome score comes as a global index, comprising of five sub-scores across emotion sub-categories of emotions: sadness, fear, anger, happiness, and love.

**Figure 10:**
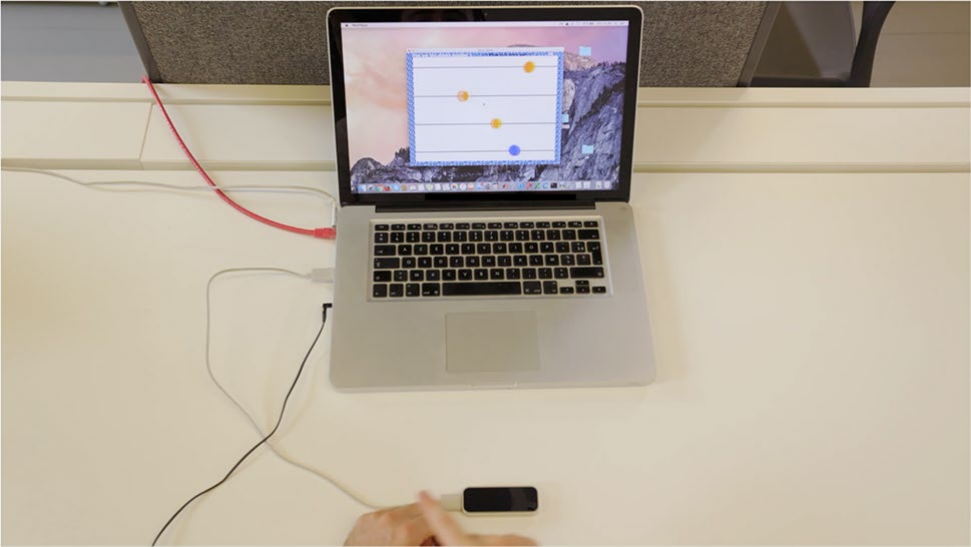
Display visible to the participant; the oscillatory movement of the preferred index finger was captured by a LEAP sensor (blue coded the moving circle of the participant in front of the screen and was always at bottom line of display, whilst the other moving circles of the other members of the same group were colour-coded as orange depicted in the upper lines of the display).

Once participants filled in the questionnaires, they were asked to launch the Chronos game on the display in their respective booths. The game started its first trial, 5 sec after all participants pressed ’START’. The goal of the game was to synchronise their motion with that of the moving circles of other players shown, representing the movements of the 3 other group members, displayed separately for each participant on their individual screen (see Fig. 10). Participants were debriefed that each of them will hear a sound early after launch of each trial, but their task is to continue to align their movement (in space and time) with the movement of others. In case they would drop their synchronisation with others, they were instructed to re-align as soon as possible to reestablish an unison movement back and forth of other moving circles.

Players were not given information of the exact topology of their interactions with others, apart from the information that all players that ’they are interacting with’ are seated in the same room. Each group performed 54 trials together, of 45 sec duration each. The trial order was fixed and preloaded to Chronos interface (this choice was required, due to technical limitations of this software not permitting for randomisation of the sound stimuli across trials). This created multiple and balanced combinations of negative, neutral and positive stimuli across participants in each group (4 participants x 18 trials per condition x 3 emotional conditions). We have decided to use a pseudo-randomised order to counterbalance the effect of order across players (Player 1—blocked design; Player 2—semi-blocked; Player 3—semi-random; Player 4—random) (see the breakdown of the trial runs between players in the Supplementary Information Table S2). After each trial participants were asked to fill in a ’pen and paper’ answer sheet with their perception of the stimuli displayed during the most recent trial. Two dimensions of the Self-Assessment Manikin (SAM), an affective rating system devised by^19^, were used to collect responses: valence and arousal (as dominance dimension was not of interest in this setup). Participants were asked to refer to graphic figurines depicting values along each of the dimensions on an interval scale (1 to 9), to indicate their emotional reaction to a stimulus presented. We obtained permission to use the SAM scale for the EnTimeMent project from CSEA media (copyright owner).

### Independent variables

Three sets of emotional sounds (pre-recorded clips) were selected from the IADS-2 battery based on their pleasure and arousal ratings (excluding sounds that were rhythmic, melodic or contained elements of speech) from the technical report^28^ for mixed-sex participants (positive valence/high arousal—9 sounds, negative valence/high arousal—9 sounds and, neutral valence/low arousal—9 sounds) (see the Supplementary Information Table S1 for the listing). Each sound was used two times per participant in order to create the required number of trials (18 per condition), constituting the three emotional conditions: (1) negative, (2) neutral and (3) positive. The time stamp of injection of the sound during the trial varied across trials between 6 and 8 sec from the trial onset (to avoid habituation and priming of participants to anticipate the sound at fixed interval for each trial) equivocally for all participants (see in the Supplementary Information Table S2).

### Dependent variables

SAM responses were anonymised and coded to match the data from PANAS and Emotional Contagion, Edinburgh handedness questionnaires. They were later merged with the performance metrics (synchronisation performance and movement speed) obtained from the LEAP sensor by participants code and group number.

### Data processing pipeline

Each group (group = 1, ..., 15) performed 54 trials in the experimental run. For each trial, the emotion of each participant and the delay of sound injection was fixed before the start of experiment, identical for all groups. The recording obtained for each participant/trial/group was a two-column matrix (script ’CHRONOS_0.m’ loads the data in the data repository). The first column was filled with time stamps going from 0 to 45 s with a frequency of 55 Hz. The second column was a recording of the position X of the index finger of a dominant hand at instant t. In order to compare data recorded across groups, trials and participants we generated a unique time vector T with frequency 100 Hz (near the double of 55 Hz as to have the least loss of information) and interpolated all X vectors (with spline method) accordingly (in order to match position X at instant t to the the X positions at interpolated instant T; and fill in any X missing data under 5 frames). These operations were realised with the script ’CHRONOS_1.m’ (see data repository).

The experimental task was to cyclically move a circle on the screen (controlled by LEAP motion sensor capturing participant’s hand position) jointly with circles representing movement of other participants in real time. To calculate the number of cycles and the mean time per cycle, we calculated in for trial the number of times the hand (number of maxima) achieved its maximal position on X axis before reversal of movement direction. Then the mean time per cycle was computed as duration of analysed segment of trial (trial total duration 45s) divided by the number of cycles. Next, for each X vector we calculated its phase with Hilbert transform (see script ”CHRONOS_2.m” in the data repository). Transformation of X vector to phase permitted to calculate synchronisation performance metrics. Herein, for a given a group and a trial the phases of 4 individuals in the group are further referenced as $\phi _{1}$, $\phi _{2}$, $\phi _{3}$, $\phi _{4}$.The Kuramoto Order Parameter opmt(t) is computed as the modulus of the mean vector of

1 $$\begin{aligned} \ opmt(t)=|\frac{1}{4} \sum _{k=1}^{4} exp(i*\varphi _k(t)) |\end{aligned}$$

at instant t in T, where ’i’ is the unitary complex number.^10^. This modulus ranges in its value between 0 and 1 and k ranges between 1 and 4. The bigger ompt(t) the stronger the phase alignment (synchronisation) between the participants. In order to extract further metrics relevant to group synchronisation performance we calculated overall synchronisation thresholds. For this, all the probability distribution was calculated of opmt of all trials and then cut into 0.25 percent quantiles. This gave the following thresholds: no synchronisation for opmt(t) the value interval [0,0.5025]; weak synchronisation ’W’ for opmt(t) in value interval [0.5025,0.6650], medium synchronisation ’M’ for opmt(t) in value interval [0.6650, 0.8274] and high synchronisation ’H’ for opmt(t) in value interval [0.8274,1] (see script CHRONOS_3.m and ancillary functions called in it, available in the data repository).

**Figure 11:**
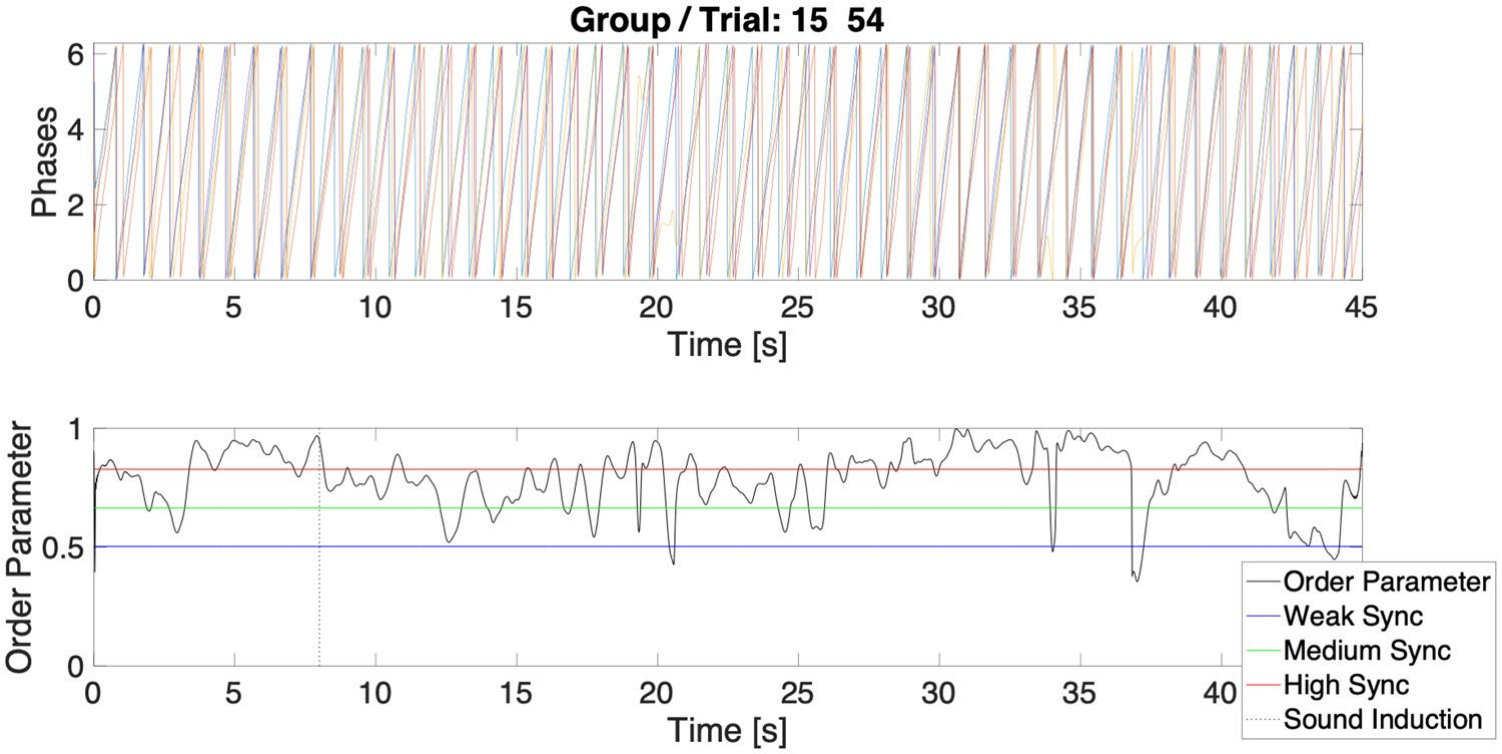
Three synchronisation bands used for the data analysis. An exemplification of the trial data—panel on the top showing phase alignment between four participants (Group 15, trial 54), the bottom panel shows the unfolding of the order parameter (opmt) over (t), across three synchronisation bands (weak, medium and weak). The dotted vertical line denotes the lag in the induction of sound (emotional induction).

Then for each threshold (W, M, H) we calculated two metric TTS - Time To Synchronisation and TIS—Time In Synchronisation. TTS is the time to surpass a given threshold of synchronisation for the first time in the selected time window for analysis and stay within for a given minimum duration (in our experiment this was empirically found to be 4,5 s). TIS was defined as the overall time duration for which opmt stayed above a given synchronisation threshold. We thus calculated for each group (trial) the values $TTS_{W}$, $TTS_{M}$, $TTS_{H}$ and $TIS_{W}$, $TIS_{M}$, $TIS_{H}$. These values were calculated for all the duration of the trial and for subsections of this interval taken for in-depth analysis—see Fig. 11. Finally, Individual Synchronisation Index (ISI) for each participant in the group was calculated separately, along with the number of cycles and time of cycle separately (for the analysis of correlation of those metrics to psychometric data). If $\phi _{g}$ notes the phase of opmt then, the relative phase of each agent k was $\phi _{k}$ - $\phi _{g}$, where ’i’ here is the unitary complex number. ISI(k) was then the mean value of moduli

2 $$\begin{aligned} \ ISI(k)= exp(i*(\phi _{k} - \phi _{g}). \end{aligned}$$

The closer ISI(k) to 1 the smaller the average phase mismatch of agent k in the group^24^. For all groups and trials, we removed from the analysis the first trial—as we found that the performance was worse than in all of the other 53 consecutive runs, which could inflate the results of Negative induction in this run (3 Negative inductions and 1 Neutral). For the descriptive statistics for the first period of all trials, across all participants, 6 sec prior to the injection of the sound is depicted below - synchronisation for the Positive (M = 0.91; SD = 0.15), Neutral (M = 0.92; SD = 0.15), Negative (M = 0.91; SD = 0.16).

We ran a linear mixed model analysis in R Studio (version 1.4.1106) using the lme4 package^51^. Significance was calculated using the lmerTest package^52^, which applies Satterthwaite’s method to estimate degrees of freedom and generate p-values for mixed models. We applied an outlier standardized removal procedure (selection of interquartile range—IQR) for the Group performance metrics to allow better fit of the LMM.

For the group variables: Order Parameter, Time in synchronisation bands, Time to reach synchronisation bands, Number of Cycles (entered into LMM as separate dependent variables), with number of positive and negative inductions per group per trial as fixed factors and Group ID as a random effect. We included Individual Synchronisation Index as the dependent variable and added fixed effects of emotion induction and interaction with Emotion Contagion Score and Sex, and included Participant ID as a random effect.

## Supplementary Information

Supplementary Information.

## Data Availability

Bieńkiewicz, M. (2023, January 9). Spook and play (data repository and codes). https://doi.org/10.17605/OSF.IO/DZWCT.
