# Supplementary material for: Impact of emotion-laden acoustic stimuli on group synchronisation performance
Source: Sci Rep. 2023 May 1;13:7094. doi: 10.1038/s41598-023-34406-2 (PMC10150690; doi:10.1038/s41598-023-34406-2)
Supplement: Supplementary file 1 — Supplementary Information. [file 41598_2023_34406_MOESM1_ESM.pdf]

Impact of emotion laden acoustic stimuli on group synchronisation performance.

Supplementary Information.

Supplementary tables

Table S1. IADS-2: ratings from the technical report[1] for the pre-selected sounds for this study.

Sound category	Sound filename	Sound ID	Pleasure Mean	Arousal Mean
NEGATIVE	ChildAbuse	278	1,57	7,27
	FemScream3	277	1,63	7,79
	Fight1	290	1,65	7,61
	Victim	286	1,68	7,88
	FemScream2	276	1,93	7,77
	MaleScream	292	1,99	7,28
	CarWreck	424	2,04	7,99
	Scream	275	2,05	8,16
	Vomit	255	2,08	6,59
NEUTRAL	Office1	320	4,23	5,48
	Pig	130	4,64	4,93
	Rain1	627	4,83	4,65
	Writing	358	4,52	4,87
	Cat	102	4,63	4,91
	Paper1	728	4,72	4,35
	BrushTeeth	720	4,86	4,18
	Panting	104	4,96	5,37
	Paint	373	5,09	4,65
POSITIVE	SportsCrowd	352	7,17	7,07
	BoyLaugh	220	7,28	6
	Party	365	6,97	6,32
	SlotMachine2	717	7,32	6,56
	Casino2	367	7,33	6,72
	Baseball	353	7,38	6,62
	Baby	110	7,64	6,03
	Crowd2	311	7,65	7,12
	Laughing	226	7,78	5,42

Note: Sound filename corresponds to the IADS-2 filename system. Colour coding denotes i.) for valence: mauve - negative; sage - neutral; Indian yellow - positive; ii.) for arousal: burnt orange - high arousal, white - medium arousal. Sounds were selected based on their pleasure and arousal rating from the technical report[1](highest valence and highest arousal scores for the positive induction; medium valence and arousal scires for the neutral induction; lowest valence and highest arousal scores for the negative induction sounds). We have excluded sounds that entailed English language (sampled from the movies, i.e., 'Attack1' 279; 'Attack2' 285) and the sounds that contained a rhythm (i.e., 'Cuckoo' 710; 'Clock' 708) or musical structure (i.e., 'Bongos' 817; 'RockNRoll' 815).

References

1. Bradley, M. M. & Lang, P. J. The International Affective Digitized Sounds (; IADS-2): Affective ratings of sounds and instruction manual. *Univ. Florida, Gainesville, FL, Tech. Rep. B-3* (2007).

Table S2. Pseudo-randomized trial order.

Trial nr.	Delay (s)	Player 1	Player 2	Player 3	Player 4
1	7	424	286	276	320
2	7	290	290	277	367
3	8	278	277	130	255
4	6	292	276	373	365
5	6	275	292	717	275
6	8	255	278	311	102
7	8	286	104	276	130
8	7	277	130	278	220
9	7	276	720	102	292
10	8	424	627	627	110
11	8	292	320	226	278
12	6	276	728	110	358
13	7	286	717	275	720
14	6	290	226	290	717
15	8	275	353	358	290
16	8	278	220	373	226
17	6	277	311	220	276
18	6	255	352	367	373
19	6	104	424	424	320
20	7	130	275	278	353
21	6	720	255	102	275
22	6	627	278	720	220
23	8	320	277	365	292
24	7	728	290	110	627
25	6	358	358	255	627
26	7	373	373	275	311
27	8	102	102	104	255
28	6	104	104	320	352
29	7	728	728	311	278
30	6	102	102	353	728
31	7	627	352	424	373
32	7	720	110	286	367
33	6	130	365	627	424
34	6	320	353	720	717
35	8	358	367	367	277
36	6	373	220	353	104
37	8	220	286	255	358
38	7	226	276	290	365
39	6	311	292	104	290
40	7	353	424	358	110
41	7	352	275	717	286
42	8	717	255	220	720
43	6	110	627	286	728
44	8	365	720	277	311
45	8	367	130	130	424
46	6	220	320	728	352
47	7	353	358	352	286
48	8	352	373	226	104
49	7	717	110	292	102
50	7	311	226	292	353
51	8	367	717	320	277
52	8	110	365	728	226
53	7	226	367	365	276
54	8	365	311	352	130

Note: Second column (Delay) indicates the time stamp (s) of a sound injection during each trial run. Each player followed a different, pseudo-randomised trial order - columns Player 1 : Player 4. The number of the sound corresponds to the IADS-2 filename system[1]. Colour coding denotes valence: mauve - negative; sage - neutral; Indian yellow - positive.

Table S3. : Median valence ratings for the sample of sounds used.

	MEDIAN NEGATIVE	MEDIAN NEUTRAL	MEDIAN POSITIVE
Valid	60	60	60
Missing	0	0	0
Mean	2.71	5.30	6.59
Std. Deviation	1.37	0.88	1.15
Minimum	1.00	3.00	3.00
Maximum	8.00	8.00	9.00

Table S4. : Median Arousal ratings for the sample of sounds used.

	MEDIAN NEGATIVE	MEDIAN NEUTRAL	MEDIAN POSITIVE
Valid	60	60	60
Missing	0	0	0
Mean	6.14	3.88	5.07
Std. Deviation	1.53	1.76	2.01
Minimum	2.00	1.00	1.00
Maximum	9.00	7.00	9.00

Table S5. : LMM table report for Period 2 - Fisher's Z score transformed median of Order Parameter

Predictors	*Estimates*	*CI*	*p*
(Intercept)	1.68	1.48 – 1.88	**<0.001**
count_neg_group [1]	0.04	-0.02 – 0.10	0.213
count_neg_group [2]	0.04	-0.00 – 0.09	0.068
count_neg_group [3]	0.06	0.02 – 0.11	**0.009**
count_pos_group [1]	0.03	-0.03 – 0.10	0.311
count_pos_group [2]	0.01	-0.03 – 0.06	0.535
count_pos_group [3]	-0.00	-0.05 – 0.04	0.933
**Random Effects**			
σ^2	0.14		
$\tau_{00\ GrNr}$	0.15		
ICC	0.52		
N_{GrNr}	15		
Observations	794		
Marginal R^2 / Conditional R^2	0.008 / 0.528		

Table S6. : LMM table report for Period 3 - Fisher's Z score transformed median of Order Parameter

Predictors	*Estimates*	*CI*	*p*
(Intercept)	1.72	1.52 – 1.92	**<0.001**
count_neg_group [1]	-0.00	-0.06 – 0.05	0.870
count_neg_group [2]	0.03	-0.01 – 0.07	0.181
count_neg_group [3]	0.06	0.01 – 0.10	**0.012**
count_pos_group [1]	0.06	-0.00 – 0.11	0.063
count_pos_group [2]	-0.03	-0.07 – 0.01	0.144
count_pos_group [3]	-0.00	-0.05 – 0.04	0.885
**Random Effects**			
σ^2	0.11		
$\tau_{00\ GrNr}$	0.15		
ICC	0.58		
N_{GrNr}	15		
Observations	793		
Marginal R^2 / Conditional R^2	0.007 / 0.579		

Table S7. : LMM table report for Period 2 - Fisher's Z score transformed standard deviation of Order Parameter

Predictors	*Estimates*	*CI*	*p*
(Intercept)	0.09	0.07 – 0.11	**<0.001**
count_neg_group [1]	-0.00	-0.01 – 0.00	0.415
count_neg_group [2]	-0.00	-0.01 – 0.00	0.231
count_neg_group [3]	-0.01	-0.02 – -0.00	**0.002**
count_pos_group [1]	-0.00	-0.01 – 0.01	0.668
count_pos_group [2]	-0.00	-0.01 – 0.00	0.233
count_pos_group [3]	-0.00	-0.01 – 0.00	0.442
**Random Effects**			
σ^2	0.00		
$\tau_{00\ GrNr}$	0.00		
ICC	0.48		
N_{GrNr}	15		
Observations	794		
Marginal R^2 / Conditional R^2	0.010 / 0.484		

Table S8. : LMM table report for Period 3 - Fisher's Z score transformed standard deviation of Order Parameter

Predictors	*Estimates*	*CI*	*p*
(Intercept)	0.09	0.06 – 0.11	**<0.001**
count_neg_group [1]	-0.00	-0.01 – 0.00	0.226
count_neg_group [2]	-0.01	-0.01 – 0.00	0.056
count_neg_group [3]	-0.00	-0.01 – 0.01	0.786
count_pos_group [1]	-0.01	-0.02 – -0.00	**0.007**
count_pos_group [2]	0.00	-0.00 – 0.01	0.323
count_pos_group [3]	0.00	-0.00 – 0.01	0.432
**Random Effects**			
σ^2	0.00		
$\tau_{00\ GrNr}$	0.00		
ICC	0.55		
N_{GrNr}	15		
Observations	794		
Marginal R^2 / Conditional R^2	0.006 / 0.550		

Table S9. : LMM table report for Time in Synchrony (H synchronisation band) in Period 2

Predictors	*Estimates*	*CI*	*p*
(Intercept)	12.39	11.15 – 13.62	**<0.001**
Count_neg_group [1]	-0.44	-0.85 – -0.02	**0.039**
Count_neg_group [2]	-0.04	-0.34 – 0.26	0.785
Count_neg_group [3]	0.29	-0.03 – 0.61	0.079
Count_pos_group [1]	-0.14	-0.55 – 0.28	0.517
Count_pos_group [2]	0.06	-0.22 – 0.35	0.660
Count_pos_group [3]	0.12	-0.18 – 0.42	0.424
**Random Effects**			
σ^2	5.22		
$\tau_{00\ GrNr}$	5.66		
ICC	0.52		
N_{GrNr}	15		
Observations	734		
Marginal R^2 / Conditional R^2	0.005 / 0.523		

Table S10. : LMM table report for Time in Synchrony (H synchronisation band) in Period 3

Predictors	*Estimates*	*CI*	*p*
(Intercept)	16.48	14.84 – 18.12	**<0.001**
Count_neg_group [1]	0.00	-0.55 – 0.56	0.992
Count_neg_group [2]	0.21	-0.18 – 0.60	0.296
Count_neg_group [3]	-0.04	-0.46 – 0.38	0.853
Count_pos_group [1]	0.56	0.00 – 1.11	**0.048**
Count_pos_group [2]	-0.38	-0.75 – 0.00	0.052
Count_pos_group [3]	-0.11	-0.51 – 0.28	0.578
**Random Effects**			
σ^2	9.73		
$\tau_{00\ GrNr}$	10.05		
ICC	0.51		
N_{GrNr}	15		
Observations	762		
Marginal R^2 / Conditional R^2	0.005 / 0.510		

Table S11. : LMM table report for Time in Synchrony (M synchronisation band) in Period 2

Predictors	*Estimates*	*CI*	*p*
(Intercept)	14.08	13.61 – 14.55	**<0.001**
Count_neg_group [1]	-0.04	-0.24 – 0.16	0.699
Count_neg_group [2]	0.01	-0.13 – 0.15	0.885
Count_neg_group [3]	0.20	0.05 – 0.35	**0.010**
Count_pos_group [1]	-0.10	-0.29 – 0.10	0.321
Count_pos_group [2]	0.09	-0.04 – 0.22	0.192
Count_pos_group [3]	0.08	-0.06 – 0.22	0.239
**Random Effects**			
σ^2	1.10		
$\tau_{00\ GrNr}$	0.79		
ICC	0.42		
N_{GrNr}	15		
Observations	697		
Marginal R^2 / Conditional R^2	0.009 / 0.423		

Table S12. : LMM table report for Time in Synchrony (W synchronisation band) in Period 2

Predictors	*Estimates*	*CI*	*p*
(Intercept)	14.89	14.83 – 14.95	**<0.001**
Count_neg_group [1]	0.02	-0.02 – 0.05	0.431
Count_neg_group [2]	0.01	-0.02 – 0.04	0.510
Count_neg_group [3]	0.04	0.01 – 0.07	**0.009**
Count_pos_group [1]	0.00	-0.03 – 0.04	0.860
Count_pos_group [2]	0.03	-0.00 – 0.05	0.055
Count_pos_group [3]	0.01	-0.02 – 0.03	0.621
**Random Effects**			
σ^2	0.04		
$\tau_{00\ GrNr}$	0.01		
ICC	0.23		
N_{GrNr}	15		
Observations	644		
Marginal R^2 / Conditional R^2	0.016 / 0.240		

Table S13. : LMM table report for Time to Synchrony (H synchronisation band) in Period 2

Predictors	*Estimates*	*CI*	*p*
(Intercept)	5.33	3.48 – 7.18	**<0.001**
Count_neg_group [1]	-0.04	-0.91 – 0.83	0.925
Count_neg_group [2]	-0.74	-1.35 – -0.13	**0.018**
Count_neg_group [3]	-0.82	-1.47 – -0.16	**0.015**
Count_pos_group [1]	0.58	-0.30 – 1.46	0.194
Count_pos_group [2]	-0.20	-0.80 – 0.40	0.516
Count_pos_group [3]	-0.36	-0.99 – 0.27	0.257
**Random Effects**			
σ^2	25.36		
$\tau_{00\ GrNr}$	12.18		
ICC	0.32		
N_{GrNr}	15		
Observations	795		
Marginal R^2 / Conditional R^2	0.012 / 0.333		

Table S14. : LMM table report for Number of Cycles completed (median) in Period 2

Predictors	*Estimates*	*CI*	*p*
(Intercept)	9.99	8.85 – 11.13	**<0.001**
Count_neg_group [1]	0.43	0.27 – 0.59	**<0.001**
Count_neg_group [2]	0.30	0.19 – 0.41	**<0.001**
Count_neg_group [3]	0.04	-0.08 – 0.16	0.500
Count_pos_group [1]	-0.37	-0.52 – -0.21	**<0.001**
Count_pos_group [2]	-0.20	-0.31 – -0.10	**<0.001**
Count_pos_group [3]	0.14	0.02 – 0.25	**0.019**
**Random Effects**			
σ^2	0.84		
$\tau_{00\ GrNr}$	5.03		
ICC	0.86		
N_{GrNr}	15		
Observations	791		
Marginal R^2 / Conditional R^2	0.032 / 0.861		

Table S15. : LMM table report for Number of Cycles completed (median) in Period 3

Predictors	*Estimates*	*CI*	*p*
(Intercept)	13.23	11.67 – 14.79	**<0.001**
Count_neg_group [1]	0.45	0.24 – 0.66	**<0.001**
Count_neg_group [2]	0.54	0.40 – 0.69	**<0.001**
Count_neg_group [3]	0.06	-0.09 – 0.21	0.448
Count_pos_group [1]	-0.41	-0.62 – -0.20	**<0.001**
Count_pos_group [2]	-0.32	-0.47 – -0.18	**<0.001**
Count_pos_group [3]	0.06	-0.09 – 0.22	0.410
**Random Effects**			
σ^2	1.50		
$\tau_{00\ GrNr}$	9.45		
ICC	0.86		
N_{GrNr}	15		
Observations	804		
Marginal R^2 / Conditional R^2	0.031 / 0.867		

Table S16. : LMM table report for Individual Synchronisation Index in Period 2

Predictors	*Estimates*	*std. Beta*	*CI*	*standardized CI*	*p*	*std. p*
(Intercept)	0.75	0.02	0.50 – 1.01	-0.12 – 0.16	**<0.001**	0.802
as.factor(relevel(EMO, ref = "Neutral"))1	0.05	0.05	0.01 – 0.10	0.01 – 0.09	**0.026**	**0.018**
as.factor(relevel(EMO, ref = "Neutral"))2	0.02	-0.02	-0.05 – 0.09	-0.08 – 0.05	0.628	0.576
EC_sum_75	0.00	0.18	-0.00 – 0.01	-0.08 – 0.43	0.185	0.185
Sex1	-0.08	0.15	-0.33 – 0.18	0.01 – 0.29	0.556	**0.037**
as.factor(relevel(EMO, ref = "Neutral"))1 * EC_sum_75	-0.00	-0.05	-0.00 – -0.00	-0.10 – -0.00	**0.046**	**0.046**
as.factor(relevel(EMO, ref = "Neutral"))2 * EC_sum_75	-0.00	-0.02	-0.00 – 0.00	-0.09 – 0.05	0.547	0.547
as.factor(relevel(EMO, ref = "Neutral"))1 * Sex1	-0.03	-0.05	-0.08 – 0.01	-0.09 – -0.01	0.154	**0.026**
as.factor(relevel(EMO, ref = "Neutral"))2 * Sex1	-0.03	-0.06	-0.10 – 0.04	-0.13 – 0.01	0.374	0.075
EC_sum_75 * Sex1	0.00	0.10	-0.00 – 0.01	-0.16 – 0.36	0.441	0.441
as.factor(relevel(EMO, ref = "Neutral"))1 * EC_sum_75 * Sex1	0.00	0.03	-0.00 – 0.00	-0.02 – 0.08	0.234	0.234
as.factor(relevel(EMO, ref = "Neutral"))2 * EC_sum_75 * Sex1	0.00	0.03	-0.00 – 0.00	-0.04 – 0.10	0.469	0.469
**Random Effects**						
σ^2	0.01					
$\tau_{00\ ParticipantNr}$	0.01					
ICC	0.35					
$N_{ParticipantNr}$	60					
Observations	3180					
Marginal R^2 / Conditional R^2	0.055 / 0.389					

Table S17. : LMM table report for Individual Synchronisation Index in Period 3

Predictors	*Estimates*	*std. Beta*	*CI*	*standardized CI*	*p*	*std. p*
(Intercept)	0.77	0.02	0.52 – 1.02	-0.13 – 0.17	**<0.001**	0.793
as.factor(relevel(EMO, ref = "Neutral"))1	0.05	0.05	-0.00 – 0.10	0.01 – 0.09	0.070	**0.015**
as.factor(relevel(EMO, ref = "Neutral"))2	0.04	-0.01	-0.04 – 0.12	-0.08 – 0.06	0.324	0.786
EC_sum_75	0.00	0.17	-0.00 – 0.01	-0.10 – 0.43	0.221	0.221
Sex1	-0.08	0.12	-0.33 – 0.17	-0.02 – 0.27	0.552	0.097
as.factor(relevel(EMO, ref = "Neutral"))1 * EC_sum_75	-0.00	-0.05	-0.00 – 0.00	-0.10 – 0.01	0.110	0.110
as.factor(relevel(EMO, ref = "Neutral"))2 * EC_sum_75	-0.00	-0.04	-0.00 – 0.00	-0.12 – 0.03	0.267	0.267
as.factor(relevel(EMO, ref = "Neutral"))1 * Sex1	-0.05	-0.03	-0.10 – 0.01	-0.07 – 0.01	0.087	0.124
as.factor(relevel(EMO, ref = "Neutral"))2 * Sex1	-0.00	-0.08	-0.08 – 0.08	-0.15 – -0.00	0.949	**0.045**
EC_sum_75 * Sex1	0.00	0.10	-0.00 – 0.01	-0.16 – 0.37	0.459	0.459
as.factor(relevel(EMO, ref = "Neutral"))1 * EC_sum_75 * Sex1	0.00	0.04	-0.00 – 0.00	-0.01 – 0.10	0.113	0.113
as.factor(relevel(EMO, ref = "Neutral"))2 * EC_sum_75 * Sex1	-0.00	-0.01	-0.00 – 0.00	-0.09 – 0.07	0.845	0.845
**Random Effects**						
σ^2	0.01					
$\tau_{00\ ParticipantNr}$	0.01					
ICC	0.41					
$N_{ParticipantNr}$	60					
Observations	3180					
Marginal R^2 / Conditional R^2	0.048 / 0.437					
